# Evaluation of an Alimentary Education Intervention on School Canteen Waste at a Primary School in Bari, Italy

**DOI:** 10.3390/ijerph17072558

**Published:** 2020-04-08

**Authors:** Nicoletta Favuzzi, Paolo Trerotoli, Maria Grazia Forte, Nicola Bartolomeo, Gabriella Serio, Domenico Lagravinese, Francesco Vino

**Affiliations:** 1 Operative Unit of Hygiene of Food and Nutrition, Department of Prevention, ASL Bari, 70122 Bari, Italy; mariagrazia.forte@asl.bari.it (M.G.F.); domenico.lagravinese@asl.bari.it (D.L.); francesco.vino@asl.bari.it (F.V.); 2 Department of Biomedical Science and Human Oncology, University of Bari Aldo Moro, 70124 Bari, Italy; paolo.trerotoli@uniba.it (P.T.); nicola.bartolomeo@uniba.it (N.B.); gabriella.serio@uniba.it (G.S.)

**Keywords:** waste food, nutritional education, waste monitoring, food quality perception

## Abstract

The “Love Food, Not Waste” project was conducted to train students on good food choices and evaluate food waste in school canteens. Teachers, parents and students were surveyed before and after training. Weights of both the served and wasted food were recorded for one week both before the educational intervention in February 2019 and after the educational intervention in March 2019, using the same menu. Students completed a food satisfaction questionnaire on the days the data were collected. For the first dish, the mean wastes per school were 1199 g before training and 1054 g after training. For the second dish, the mean wastes per school were 246 g before training and 220 g after training. For the side course, the means wastes per school were 663 g before training and 747 g after training. The results did not significantly differ among weeks or schools. Less food was wasted when boys judged the food’s general aspects like smell, taste and appearance as positive; more food was wasted when girls judged these factors as negative. Food waste monitoring is mandatory but does not always occur. Analyzing food waste relative to students’ food perceptions can help determine whether educational interventions can help reduce waste. Students’ satisfaction must also be considered.

## 1. Introduction

As the population grows worldwide, one of the most worrying paradoxes is the increased amount of food waste produced globally. In the USA, both food consumption and individuals’ weights have progressively increased, while the amount of food wasted is estimated to equal the amount of food consumed [1].

A study entitled “Global Food Losses and Food Waste” conducted by the FAO (Food and Agricultural Organization) found that between August 2010 and January 2011, approximately 1.3 billion tons of food were wasted annually worldwide, of which 80% were still consumable at the time they were wasted [2].

In low-income countries, more than 40% of food losses occur during the post-harvest and processing phases because of the lack of infrastructure. In industrialized countries, more than 40% of food losses occur during retail and consumption. The per capita food loss in Europe and North America is 280–300 kg/year from a production of 900 kg/year. In sub-Saharan Africa and South/Southeast Asia this loss is 120–170 kg/year from a production of 460 kg/year. Per capita food wasted by consumers in Europe and North America is 95–115 kg/year, while in sub-Saharan Africa and South/Southeast Asia this loss is only 6–11 kg/year. The total food waste in industrialized countries (222 million tons) nearly equals the total net food production in sub-Saharan Africa (230 million tons). It was also estimated that food waste results in a waste of resources equal to approximately 250,000 billion liters of water and 1.4 billion hectares of soil. The amount of food waste also results in approximately 3.3 billion tons of CO_2_ being released into the atmosphere annually. Regarding economic impacts, the economic value of food wasted globally is estimated at around 1000 billion dollars/year (approximately 900 billion euros/year), in addition to 700 billion dollars in environmental costs (approximately 600 billion euros/year) and 900 billion dollars in social costs (approximately 800 billion euros/year) [3].

A new FAO report in 2019 distinguishes between food loss, defined as the decrease of quantity caused by the action of all actors in the food supply chain excluded retail, and food waste, defined as the decrease in quantity by the actions of retailers, food services and consumers. A 13.8% food loss was reported. At the consumer level the FAO report showed the results of many studies stating that food waste was in the 14–37% range for perishable animal product and 9–20% for fruits and vegetables. Even if consumers are aware of the food waste problem, households produce an amount of food waste equal to 9% of the per capita expenditure for food [4]. Thus, reducing food waste would help conserve natural and social resources and the distinction between loss and waste should help focus on the analysis of the different stages of the food supply chain: what happens from harvest to post-harvest and at the consumer level, retail and services.

At the consumer level four aspects were identified as food waste drivers: stocking too much food, cooking too much, leaving on dishes, and spoilage of unconsumed prepared food. Each of these steps contributes to food and resources waste, but this seems to not be perceived by households, even in middle income countries [5]. On the other hand a study by Aschemann-Wizel showed that factors such as value consciousness and social norms had a slight effect on food waste at home, resulting in more effective behavior, consumers’ convenience and deal proneness [6].

Food services, on the contrary, pay more attention to financial losses, therefore the amount of food waste and losses appear low, because the responsibility has shifted to the consumer [7]. In any case, in the European Union, at all stages of the food chain from production to consumption, approximately 100 million tons of food are wasted annually, of which 14% is attributed to collective catering [8]. Schools catering, among institutional ones, should be investigated, because they offer an opportunity to evaluate factors related to food waste and loss, as well as, more importantly, to increase awareness about sustainability and change behaviors [7].

In Italy, an investigation by the Waste Watcher Observatory of Last Minute Market found increased perceptions of waste and increased beliefs that the fight against food waste must start in schools, as 53% of Italian students have lunch at school in kindergarten, primary and secondary schools [9]. The National Food Waste Prevention Plan, launched by the Ministry of the Environment in 2014 as priority actions against waste, introduced training courses in schools at all levels regarding food, environmental education and food waste [10].

Italian laws enable collecting and donating food and surplus food to account for the issue of food waste (Law 116/2016 “Anti-waste law”; Legislative Decree n.50 of 18 April 2016 New Procurement Code) as well as regional acts and regulations at local levels for the same purpose (regional guidelines for school and corporate catering in school catering). These measures promote equity and sustainability while maintaining the principles of food safety [11].

Many studies underline the role of educational campaign to improve awareness and change behaviors, as example a study on nutritional education found that information education raise awareness and it shown a negative correlation with plate waste rates [12] and another study in South Korea shown that students who recognized the need for nutritional education were significantly lower and students’ attitudes towards food waste reduction were negatively correlated with plate waste rate [13].

In this complex context it was started the “Love Food, Not Waste” (LFNW) project that aims to promote a participatory programming vision of lifestyles, with active involvement by school managers, teachers, students, parents and food providers with the possibility of alliances with other public and private subjects, to plan evidence-based prevention interventions according to the principles of effectiveness and sustainability of public action. One of the project’s objectives is to evaluate the food waste in school canteens in Bari. This work shows the results of food waste measurements, more specifically plate waste, before and after educational interventions aimed at improving awareness of consumed foods.

## 2. Materials and Methods 

The LFNW project promoted an educational intervention for students through a press release posted on school websites, starting in January 2019 and ending in May 2019. This included training for teachers, canteen employees and parents. The food waste was detected and weighed before and after the intervention.

School executives from 13 educational districts in the metropolitan area of Bari were invited to an informational meeting, where the project was presented with a contextual request for membership. All the invited schools joined the project, although one dropped out during the first survey because of logistical difficulties. Thus, 12 schools were finally enrolled, including 361 third-grade students.

Meals were prepared in the single cooking center of the catering company that serves all the primary schools of the metropolitan area of Bari. Food is delivered at each school by mixed thermal (heat/cold) carts with multiportions pan and distributed by means of graduated ladle. The weight of served food is determined as the product of the number of pupils present and the weight of a standard portion, based on the nutritional needs established by regional guidelines [11], placed on the dish using a standardized graduated ladle. The portion size was equal for males and females: 200 g for the first course, 60 g for the second course, 100 g for the side course. Waste and surpluses were observed and weighted at the end of each meal: the discarded food was placed in bags and weighed on a kitchen scale separately for each course. The administrative staff and teachers in charge weighed the food waste, allowing possible collaboration from the students. The first round of food waste detection was conducted daily for 5 days between February 18 and February 25, 2019. The second survey was conducted from March 18 to March 22, 2019, with the same menu provided during the previous survey.

The data were collected using different forms for each participant group: canteen attendants completed a form with information on the time the meals arrived and the time the meal service started and ended; teachers completed a form with information on the number of children present on that date, the weight of the meals in the served dishes, the number of meals eaten in their entirety, the number of those wholly discarded (through visual evaluation), and the total weight of the meals partially or completely unconsumed; the students completed a questionnaire on the perceived overall quality of the meal and their perceptions of the appearance, smell, and taste using an analog scale expressed by smiley emoticons representing three possible answers: unpleasant, pleasant, and very good.

Sixty-nine teachers who participated in the training and collaborated in the educational activities were chosen from all health referents and those who accompanied the students during meals and ate the same meals as the students.

Twelve of the canteen employees who distributed the meals and five supervisors provided by the management company attended the informational meetings and administered the surveys.

The teachers and canteen staff were separately trained on the project’s rationale, objectives, and implementation methods, focusing on the methods used to weigh the waste and the correct way to fill out the survey forms and questionnaires.

The teachers were trained on food safety and nutrition issues, the epidemiology of disorders related to poor nutrition in children, data on food waste and its ethical, environmental and economic implications. These issues were then addressed in class to introduce the project to the students and implement the teachers’ tutoring roles during classroom sharing and brainstorming.

The canteen staff were trained on administration and school lunch management as per current legislation. The parents were taught the epidemiology and clinical consequences of incorrect nutrition in children, the characteristics of a balanced food day and conscientious spending.

For the educational intervention aimed at children, an innovative teaching method known as a flipped classroom (upside-down class) was used, with the first moment consisting of autonomous learning by each student at home. The second step involved putting the new knowledge into practice under the teachers’ guidance [14].

Four “Didactic Sheets” were formulated: “Cereal and legumes”, “Milk and derivatives”, “Fruits and vegetables” and “Fish”, in which various perspectives and critical and important food groups in children’s nutrition were analyzed: production and selling methods, how to choose and evaluate food quality, conservation methods, cooking methods, nutritional contents and recommended consumption frequencies. These teaching cards were given to children to be studied at home, possibly with the family, considering that the active and participatory collaboration of families in the food education activity constitutes a driving element for its success. Furthermore students should experience with parents active participation in purchasing, reading labels, looking and touching foods to evaluate shape, consistency, and differences among different foods. Then they should experience, still at home with parents, taste of food and learn how to preserve, to cleanse and to cook. All these practical experiences should be reported in modules as homework.

In the second step teachers evaluated the acquired skills by children in the classroom, through discussions and brainstorming. Then a new experience of food and meals was done at practical level in the canteen, under the guidance of the trained teachers.

### Statistical Analysis

Both the weights and percentages of the food waste to served food for each day and each school and for the first, second and side courses were analyzed. The percentage of waste was calculated as the sum of the weight of the waste per school for each day divided by the total weight served in the same school and day.

Both the weight and percentage were normally distributed, as verified by the Shapiro-Wilks test, and were reported as the average and standard error estimated by linear models.

To assess the effect of educational intervention on weight and waste percentage, a mixed-effect linear model was applied that accounted for repeated measurements in the schools. The first level unit was each day both before and after the intervention and the second level unit was each school. The school and educational interventions were considered fixed effects too.

For the first level unit (each day of data collection) *i* = 1 to 5, and the second level unit (each school) *j* = 1 to 12,

Model 1 was:

(1) $$y_{ij}=\beta_{0j}+\cdot \sum^{\;}\beta_{m}\cdot x_{ijm}+\cdot \varepsilon_{ij}$$

where β_0j_ is the average intercept γ_00_ plus group-dependent deviation U_0j_, and for *m* = 1 to 2, are the regression coefficients for the variables: m = 1 school, m = 2 data before or after the intervention. Model (1) was applied for independent weight (1a) and then for independent percentage of discarded food (1b).

The results are reported as the mean and standard error derived from the model estimates. To evaluate the statistical significance of factors in the model the F test for type III variance estimation methods was used. The p-value was adjusted for the multiplicity of the comparison between schools as per the Tukey method.

The relationship between children’s judgments and the discarded meal quantity, both in weight and percentage, was determined using a mixed-effect, generalized linear model (Model 2).

For the first level unit (each day of data collection) *i* = 1 to 5, and the second level unit (each school) *j* = 1 to 12,

Model 2 was:

(2) $$y_{ij}=\beta_{0j}+\cdot \sum^{\;}\beta_{m}\cdot x_{ijm}+\cdot \varepsilon_{ij}$$

where β_0j_ is the average intercept γ_00_ plus group-dependent deviation U_0j,_ and for *m* = 1 to 7, are the regression coefficients for the variables: *m* = 1 number of males who judge food as unpleasant, *m* = 2 number of males who judge it as pleasant, *m* = 3 number of males who judge it as very good, *m* = 4 number of females who judge the food as unpleasant, *m* = 5 number of females who judge it as pleasant, *m* = 6 number of females who judge it as very good., *m* = 7 dichotomic variables that indicates the educational treatment.

For each outcome (the weight, Model 2a, and percentage, Model 2b, of food waste), a model was estimated for each dimension of quality judged by the children (appearance, flavor, smell and overall judgment). The independent variables were the effect of the educational intervention (entered as dummy variable: 0 = before, 1 = after) and the number of boys and girls who rated the meal on a three-level scale (very good, pleasant, unpleasant). The model also assessed the school, the time elapsed between the arrival and the administration of the meal, and the duration of the administration. Goodness of fit was assessed using the Akaike information criterion and deviance information criterion values, with lower values being better. The p-value of each variable entered in the model was used to evaluate the final model applied for each course (first, second and side courses). The model allowed estimating the quantity in grams wasted for each child that formulated a judgment. The same procedure was followed for the model with the percentage of waste as the dependent variable.

Possible relationships between the weight of the food served, the number of second servings and the amount wasted for each dish were assessed with the Spearman correlation coefficient. A nonparametric test was used because the number of dishes served was not normally distributed. Statistical significance was considered at *p* < 0.05. All analyses were conducted using SAS 9.4 software (SAS Institute, Cary, NC, USA) [15].

## 3. Results

The number of children who participated each day ranged from 257 (F:114; M:143) to 323 (F:155; M:168), with an average of 16 per class, a daily range between four and 25, and a total of 3393 meals served, in addition to 70 second servings of the first course, 278 second servings of the second course and 109 second servings of the side course. The weight of waste of the first courses (Table 1) before the educational intervention per class and day averaged 1199.31 g (standard error (SE), 96.8 g). After the educational intervention, the average was 1054.8 g (SE, 89.8 g), but this difference was not statistically significant. The average percentages of daily waste per class were 35% (SE, 2.7%) and 34% (SE, 2.5%) before and after the educational intervention, respectively.

The weights of the discarded food waste from the second course (Table 2) averaged 246.9 g (SE, 34.2 g) and 220.9 g (SE, 31.7 g) before and after the educational intervention, respectively. The average percentage discarded decreased slightly between the two surveys: the first was 32% (SE, 4%); the second was 31% (SE, 4%). These differences were not statistically significant.

The side dishes were the most discarded (Table 3); 663.4 g (SE, 54.6 g) of food were discarded before the educational intervention and 747.9 g (SE, 50.6 g) of food were discarded after the intervention. The percentages before and after the surveys were 55.4% (SE, 4.1%) and 59.7% (SE, 3.8%), respectively. Although the differences were not statistically significant, the side dish was the only dish for which the amount of food waste increased.

The correlation between the waste from a course and its quantity served or the quantity served from the previous course (Table 4) was directly proportional to the rejected portion of the first course and the number of portions of the first course served (rs = 0.52, *p* < 0.001). For the second dish, the additional portions served were directly proportional to the percentage of food waste (rs = 0.19, *p* < 0.0001). This relationship was also observed between the amount served and the amount discarded (rs = 0.41, *p* < 0.0001). The side dish followed the same trend as the second course with direct relationships between the served weight and waste weight (rs = 0.24, *p* < 0.0001), between the percentage of the weight served and the percentage of waste weight (rs = 0.27, *p* < 0.0001), and between the additional portions and percentage of side course rejection (rs = 0.24, *p* < 0.0001). The percentage of additional portions of the first and second course that were rejected was weakly directly proportional (rs = 0.19, *p* < 0.0001) to the weight of the side course waste (rs = 0.15, *p* = 0.0023). The additional second course portions were also weakly directly proportional to the weight of the side course waste (rs = 0.15, *p* = 0.0022).

The influence of meal acceptance on the quantity and portion of rejected food was assessed through a regression model that allowed highlighting the quantity (Model 2a) and the percentage (Model 2b) of the food rejected for each boy and girl and the level of satisfaction (Table 5). For the first course, the waste increased significantly when both boys and girls judged the food as unpleasant. When the overall perception of the meal was judged as unpleasant by girls, the waste weight increased by 142.1 g (SE, 19.8 g, *p* < 0.0001), and by boys the waste weight increased by 56.6 g (SE, 20.6 g, *p* = 0.0063). When girls judged the food as “pleasant”, the waste increased by 65.3 g (SE, 24.7; *p* = 0.0085), but when boys judged it as “very good”, the waste decreased by 52.5 g (SE, 16.9, *p* = 0.0021) per student. When considering children’s judgments, the educational intervention reduced the waste weight from the first course to 152.5 g (72.1 g; *p* = 0.035). Analysis of the meal characteristics yielded similar conclusions: if the appearance, taste, and smell were unpleasant, the food wasted was respectively increased by 132.3 g, 150.8 g and 115.4 g for appearance, taste and smell for girls and by 46.2 g, 62.8 g and 63.2 g for appearance, taste and smell for boys. Neutral judgments (pleasant) yielded increased waste weights for both boys and girls.

Unpleasant and pleasant judgments expressed by both boys and girls resulted in significantly increased food waste for the second courses (Table 6). For each girl who expressed a negative judgment, the waste increased by 37.5 g (SE, 7.5 g; *p* < 0.0001); for each boy, the increase was 35.6 g (SE, 7.8 g, *p* < 0.0001). For the second course, the waste was statistically significantly reduced by 11.9 g (SE, 5.6 g, *p* = 0.033) when girls expressed a “very good” judgment. The same results were obtained for appearance, taste and smell. The effect of the educational intervention was not statistically significant, although the regression coefficient was negative, suggesting that waste from the second course was reduced after the educational intervention (-41.5 g, SE = 25.1 g, *p* = 0.0988).

Side courses were unassociated with judgment in some cases. When boys judged the food as unpleasant, the waste was significantly increased (+32.5 g, SE: 12.8; *p* = 0.0115); when boys judged the food as “very good”, the waste was significantly decreased (−54.7 g, SE: 59.9 g; *p* = 0.0002; Table 7). When girls rated the food as pleasant, the food waste was significantly increased (+46.2 g, SE: 16.8 g, *p* = 0.0064), suggesting that the difference in food waste for girls is not connected to their judgment of the food for the side course. Notably, the educational intervention was irrelevant here: the weight discarded was reduced by 24.9 g (SE: 58.9; *p* = 0.6734), which was not statistically significant. Similar results were obtained for other aspects of side course quality.

## 4. Discussion

This study revealed a high amount of food waste in school canteens and that a single educational intervention, however complex, is insufficient to observe significant changes. In 2015, the Ministry of Health in collaboration with the Ministry of Education and Research launched an initial fact-finding survey on school catering to outline the overall situation and stimulate its constant improvement. The Ministry of Health survey, to which 15% of all institutes responded (1168 SU 7733), showed that more than half of the schools did not monitor waste and had no monitoring procedures. In the schools that monitored food waste, it was monitored mainly by the staff that distributed the meals. The survey concluded that each school must monitor food surpluses and waste and determine the causes to reduce waste and reuse food [9]. The LFNW project aimed to measure waste and assess its causes relative to students’ satisfaction.

Although high, the total waste found in our study was consistent with that of other Italian studies: in a study conducted in Bologna schools, the food waste was near 30%, with an important difference in side dishes (58%) [16], and a study conducted in Pistoia canteens found a waste of 40% and was also more focused on side dishes [17]. A study in Rome found a 37% difference [18]. Studies in other countries have also yielded concerning results: an American study detected a 34% difference [19], and a Swiss study detected a 23% school food mix [20].

A study conducted in primary schools in Barcelona showed a mean discarded weight 21–47 g as plate waste. The lower values were observed where there was an increased awareness of pupils due to two main factors: the inclusion in the students’ career eating behaviour and patterns and a very attention of school managers and teachers to sustanaibility [7]. This observation is the main reason to move our project towards the raise of a cultural approach in teachers, pupils and parents. The enthusiastic participation of children appear to be not sufficient to see an effect after only one educational intervention. Even if the flipped-classroom approach involve parents and children in a pragmatic learning, based on experience and despite of parents participation, once ended the school work, both parents and children return to their usual habits. This hypothesis could explain the slight and not significant difference of waste before-after the intervention.

Our study was conducted in schools served by an external catering service, in which the time that the food was served was not correlated with the difference. However, other studies have found that 42% more food is wasted in schools that provide external catering compared with schools with internal cooking centers [20]. On the other side the food waste audit in Florida shown that the food waste was less in the school that had external service [21]. The differences between canteens with external or internal cooking centers is an interesting argument, because the study in Florida shown more waste when there is an internal cafeteria or an internal cooking center. The study by Darqui et al. instead highlight the advantage to have an internal service, if students are involved in the canteen [7].

Notably, the dietary tables of the Bari public schools (the current specifications have been in force since the beginning of the 2018/2019 academic year) have recently been adapted to the ministerial guidelines [22], which follow regional guidelines [11] adhering to the updated indications regarding LARN and food consumption frequencies. Compared with previous menus, choices have been pursued to eliminate industrial products (fish sticks, duchess potatoes), introduce foods such as eggs, and provide correct combinations to favor a balance between macronutrients and better absorption. Specifically, cereals other than wheat and whole grains (spelt, whole wheat pasta and bread) have been added, the frequency of consumption of some foods, such as legumes, has been increased, seasonality, organic and zero km requirements for fruit and vegetables have been included, and different cultural foods (such as cous cous) have been added to transmit a message of interculturality. In the study by Wilkie et al. the amount of fruit and vegetables waste, often the most wasted, and its changes after the introduction of the Healthy Anger-Free Kids Act, that requires fruit to be served to each kid, was highlighted – this could increase food waste and demands solutions [23].

Reduced waste, in any case, does not always indicate a nutritionally balanced and adequate meal; for example, of 120 Danish primary schools, only 19% comply with the guidelines for healthy canteen food compared with a careful 73% waste reduction [24]. An awareness campaign to reduce waste at a Portuguese university reduced waste by 15%, but without offering effective nutrition to the students [25].

Our study has shown that judgement on meals has a great impact in primary school kids. Other studies shown their selectivity [26]. If a pupil judge as unpleasant the amount of weights and leftovers increase (both in weight and in percentage). Offering healthy but less palatable food, therefore, presents the risk of greater waste. Meals served in a middle American school with increased vegetable quantities relative to self-service showed an increased waste of 48–74% vs 17–30% [23,27]. A study by Derqui shown that appearance could be a significant factors to increase waste, expecially for fruits that should “appear perfect” [7]. We couldn’t evaluate fruits because it happens that kids consume it later in the afternoon. Vegetables, instead, are part of side course and they were wasted or discarded as observed in previously cited studies.

Judgement on meals was not the only possible cause of the food waste in our study. The results have shown that among females the amount of waste increases even if they express a positive judgement. This could due to the portion size: males and females received portions of equal size distributed by the external service staff using a graduated ladle. Therefore the increase of waste could be due to the surplus in the dishes of female. Furthermore there is a positive relation between waste and served weight and number of additional portions. Pupils ask for more portions and teachers and service staff gave them, but probably not all additional portions were effectively consumed. Steen et al. analysed with a regression model the factors causing food waste and the most important was portion size independently of sex [21]. In this study another significant factor was the type of kitchen, but in our work we can’t comment on this, because schools are served by an external catering service, managed by a sole company for all the schools of the city.

A factor that could indirectly increase food waste could be the setting in which food is served, if rooms in which meals are consumed not apt for the purpose: if the service is too fast and the necessity for teachers to go back to classroom could all have an effect on kids. In our study these factors were analysed anyway and resulted not significant, therefore they were removed from the model. The previous cited study by Steen did not show an effect of the settings because this seem correlated with the main factors: age, kitchen and portion size [21].

The LFNW project presented here had the ambitious goal of training and educating primary school teachers, students and parents to consume food conscientiously and reduce food waste. The Whole School Approach shows that the school and healthcare systems have fundamental common interests that combined can enable schools to become place to learn, work and live better while also becoming healthier. The “Schools that promote Health” (School for Health in Europe – SHE) program supports better teaching and learning processes and work together with the community to strengthen social capital and health literacy. In planning its improvement, a school that promotes health must adopt a holistic approach that includes both food and environmental education, aimed at all students, their families, teachers, and non-teaching staff, to develop skills in all members of the school community and improve the physical and social environment, widen their territory and strengthen collaboration with the local community [28]. All studies converge on the positive effect of promotion of the awareness of ethic principle of food sustainability [7,21,23]

Several studies have highlighted the need to improve food waste data collection [16,17,29,30,31] and implement an integrated intervention that provides food education and develops awareness of food and waste [32]. Our study did not give any insight into the catering service, but interventions to prevent waste and reuse and recover surpluses, which involve all food service restaurant employees, are also needed [33].

## 5. Conclusions

This study failed to find a robust effect of educational intervention on the amount of waste produced in school canteens. Food education likely encouraged students to taste dishes that they would not otherwise have eaten, but the appearance, smell and flavor matter greatly. The relationship between waste and wait times and meal consumption was weak, which could have influenced the organoleptic aspects, while the weights of the food served and the amount of subsequent dishes discarded affected the waste.

Consuming meals at school enables food education, which may affect students’ knowledge of the dishes, their ingredients and preparation, thus making students more aware of their choices.

The waste detection methods in this experiment were difficult for organizational reasons, suggesting that although waste monitoring is recommended, it is difficult to manage without service personnel assistance. Greater parental participation is also desirable.

Despite these considerations the participation of kids and teachers was good, therefore more work should be done to increase knowledge and awareness about the themes of healthy nutrition and food sustainability.

Projects such as the “Love Food Not Waste” project deserve greater attention and participation to approach participatory food education that will enable food balancing and waste reduction in future generations.

## Figures and Tables

**Table 1:** Weights and percentages of students’ food waste from the first course before and after educational intervention. No statistically significant differences were found between the 2 time points or between schools.

	Weight of Food Waste (in Grams)				Percentage of Food Waste			
	Before Educational Intervention		After Educational Intervention		Before Educational Intervention		After Educational Intervention	
School	Mean	Standard Error	Mean	Standard Error	Mean	Standard Error	Mean	Standard Error
1	983.2	480.9	1232.5	407.1	53.2%	13.4%	61.5%	11.3%
2	484.0	445.9	488.9	433.2	22.4%	12.4%	23.6%	12.1%
3	837.3	326.9	422.2	296.6	37.7%	9.1%	17.9%	8.3%
4	1739.2	173.3	1393.3	154.9	45.3%	4.8%	38.9%	4.3%
5	1581.1	312.2	1119.7	285.5	30.5%	8.7%	22.2%	7.9%
6	599.3	315.3	644.0	294.1	30.3%	8.8%	30.3%	8.2%
7	1311.5	234.4	350.3	211.6	32.7%	6.5%	9.9%	5.9%
8	1805.4	357.1	1385.9	348.2	41.7%	9.9%	42.0%	9.7%
9	645.6	475.4	1246.8	459.9	23.0%	13.2%	50.8%	12.8%
10	1079.2	263.7	1032.9	256.6	34.0%	7.3%	37.0%	7.1%
11	1471.9	218.6	1464.8	199.8	29.6%	6.1%	32.2%	5.6%
12	1853.9	244.0	1872.3	210.2	40.1%	6.8%	40.6%	5.8%
**Total**	**1199.3**	**96.8**	**1054.5**	**89.8**	**35.0%**	**2.7%**	**34.0%**	**2.5%**

**Table 2:** Weights and percentages of students’ food waste from the second course before and after educational intervention. No statistically significant differences were found between the 2 time points or between schools.

	Weight of Food Waste (in Grams)				Percentage of Food Waste			
	Before Educational Intervention		After Educational Intervention		Before Educational Intervention		After Educational Intervention	
School	Mean	Standard Error	Mean	Standard Error	Mean	Standard Error	Mean	Standard Error
1	162.6	169.9	226.1	143.9	28.8%	21.9%	35.3%	18.6%
2	73.4	157.6	60.4	153.1	6.3%	20.3%	7.3%	19.8%
3	205.8	115.6	125.9	104.9	29.9%	14.9%	18.6%	13.5%
4	321.9	61.3	333.6	54.8	47.2%	7.9%	47.3%	7.1%
5	405.8	110.4	250.3	100.9	51.1%	14.2%	32.4%	13.0%
6	106.7	111.5	102.6	103.9	14.3%	14.4%	16.1%	13.4%
7	304.7	82.9	26.5	74.8	36.9%	10.7%	3.7%	9.7%
8	464.1	126.2	152.6	123.1	38.9%	16.3%	18.2%	15.9%
9	34.1	168.1	210.6	162.6	4.7%	21.7%	29.9%	21.0%
10	248.9	93.2	294.0	90.7	35.7%	12.0%	43.1%	11.7%
11	327.7	77.3	393.9	70.6	45.2%	10.0%	54.7%	9.1%
12	308.0	86.3	474.9	74.3	47.0%	11.0%	69.8%	9.6%
**Total**	**246.9**	**34.2**	**220.9**	**31.7**	**32.0%**	**4.0%**	**31.0%**	**4.0%**

**Table 3:** Weights and percentages of students’ side dish waste before and after educational intervention. No statistically significant differences were found between the 2 time points or between schools.

	Weight of Food Waste (in Grams)				Percentage of Food Waste			
	Before Educational Intervention		After Educational Intervention		Before Educational Intervention		After Educational Intervention	
School	Mean	Standard Error	Mean	Standard Error	Mean	Standard Error	Mean	Standard Error
1	495.6	271.1	959.8	229.5	44.2%	20.5%	76.2%	17.3%
2	502.0	251.4	667.5	244.1	41.9%	19.0%	52.5%	18.5%
3	738.0	184.3	426.0	167.2	67.1%	13.9%	35.9%	12.6%
4	796.9	97.7	835.2	87.4	67.3%	7.4%	71.8%	6.6%
5	805.4	175.9	1148.1	160.9	73.4%	13.3%	87.9%	12.2%
6	308.4	177.7	532.0	165.8	25.0%	13.4%	44.5%	12.5%
7	72.3	132.1	17.1	119.3	6.6%	10.0%	1.0%	9.0%
8	933.3	201.2	907.9	196.3	65.0%	15.2%	67.8%	14.8%
9	759.1	267.9	825.4	259.3	61.9%	20.3%	61.1%	19.6%
10	558.2	148.6	661.3	144.7	50.1%	11.2%	56.4%	10.9%
11	709.3	123.2	1072.4	112.6	61.8%	9.3%	85.0%	8.5%
12	1282.5	137.54	922.9	118.5	100.5%	10.4%	75.8%	9.0%
**Total**	**663.4**	**54.6**	**747.9**	**50.6**	**55.4%**	**4.1%**	**59.7%**	**3.8%**

**Table 4:** Correlation coefficients (*p*-values) of the associations between waste weight, percentages of waste and number of additional served dishes for each course.

Variables	First Course—Served Weight	First Course—Number of Additional Served Dishes	First Course—Wasted Weight	First Course—Percentage of Waste	Second Course—Served Weight	Second Course—Number of Additional Served Dishes	Second Course—Wasted Weight	Second Course—Percentage of Waste	Side Course—Served Weight	Side Course Number of Additional Served Dishes
First course—Wasted weight	0.21 *(<0.0001*)	0.31 *(<0.0001*)								
First course—Percentage of waste	0.24 (*<0.0001*)	0.53 (*<0.0001*)	0.87 (*<0.0001*)							
Second course—Served weight	0.62 (*<0.0001*)	0.09 (*0.0487*)	0.36 (*<0.0001*)	0.07 (*0.167*)						
Second course—Number of additional served dishes	0.23 (*<0.0001*)	0.09 (*0.058*)	0.25 (*<0.0001*)	0.13 (*0.0101*)	0.44 (*<.0001*)					
Second course—Wasted weight	0.34 (*<0.0001*)	0.21 (*<0.0001*)	0.27 (*<0.0001*)	0.08 (*0.0842*)	0.41 (<.0001)	0.05 (*0.2706*)				
Second course—Percentage of waste	0.15 (0.0032)	0.19 (<0.0001)	0.12 (0.0126)	0.04 (*0.4296*)	0.09 (*0.054*)	0.19 (<*0.0001*)	0.92 (<*0.0001*)			
Side course—Served weight	0.71 (<*0.0001*)	0.17 (*0.0006*)	0.34 (<*0.0001*)	0.002 (*0.965*)	0.47 (<*0.0001*)	0.17 (*0.0004*)	0.18 (*0.0003*)	0.003 (*0.9528*)		
Side course—Number of additional served dishes	0.17 (*0.0005*)	0.13 (*0.0082*)	0.06 (*0.246*)	0.03 (*0.5034*)	0.18 (*0.0002*)	0.17 (*0.0008*)	0.09 (0.0733)	0.02 (*0.7327*)	0.14 (*0.0054*)	
Side course—Wasted weight	0.12 (*0.0129*)	0.15 (*0.0023*)	0.27 (*<0.0001*)	0.18 (*0.0002*)	0.17 (*0.0007*)	0.15 (*0.0022*)	0.28 (<*0.0001*)	0.22 (*<0.0001*)	0.24 (<*0.0001*)	0.15 (*0.0019*)
Side course—Percentage of waste	0.24 (*<0.0001*)	0.05 (*0.3454*)	0.08 (*0.1147*)	0.17 (*0.0004*)	0.14 (*0.0338*)	0.06 (*0.221*)	0.14 (*0.0042*)	0.19 (*<0.0001*)	0.27 (*<0.0001*)	0.24 (*<0.0001*)

**Table 5:** Regression coefficients of the model evaluating waste weights and percentages from the first course for numbers of boys and girls expressing pleasant, indifferent or unpleasant judgments on overall aspect, appearance, smell and taste.

	FIRST MAIN COURSE			
	WEIGHT		PERCENTAGE	
Overall Aspect	β (SE)	*p*-Value	β (SE)	*p*-Value
Intercept	648.3 (161.5)	0.002	42.0% (5.0%)	<0.0001
Girls, unpleasant	142.1 (19.8)	<0.0001	1.8% (0.6%)	0.0024
Girls, pleasant	65.3 (24.7)	0.0085	0.7% (1.0%)	0.3511
Girls, very good	9.3 (15.2)	0.5423	−1.0% (0.4%)	0.0043
Boys, unpleasant	56.6 (20.6)	0.0063	2.0% (0.6%)	0.0058
Boys, pleasant	83.6 (23.9)	0.0005	1.0% (1.0%)	0.1334
Boys, very good	−52.5 (16.9)	0.0021	−3.0% (0.5%)	<0.0001
After educational intervention vs before	−152.5 (72.1)	0.035	−5.0% (2.0%)	0.0274
**Appearance**				
Intercept	707.5 (160.2)	0.001	44.0% (5.0%)	<0.0001
Girls, unpleasant	132.3 (21.1)	<0.0001	2.0% (0.6%)	0.0109
Girls, pleasant	89.6 (22.4)	<0.0001	1.0% (1.0%)	0.2821
Girls, very good	16.6 (15.3)	0.2763	−1.0% (0.5%)	0.0286
Boys, unpleasant	46.2 (23.2)	0.0477	2.0% (1.0%)	0.0298
Boys, pleasant	55.3 (22.5)	0.0146	0.3% (1.0%)	0.645
Boys, very good	−64.7 (17.9)	0.0004	−3.0% (0.5%)	<0.0001
After educational intervention vs before	−126.9 (73.4)	0.0848	−4.0% (2.0%)	0.086
**Taste**				
Intercept	665.3 (160.8)	0.0016	42.0% (5.0%)	<0.0001
Girls, unpleasant	150.8 (19.9)	<0.0001	2.0% (0.6%)	0.001
Girls, pleasant	54.1 (24.9)	0.0313	1.0% (1.0%)	0.3513
Girls, very good	14.1(14.8)	0.3405	−1.0% (0.4%)	0.0065
Boys, unpleasant	62.8 (20.4)	0.0023	2.0% (1.0%)	0.0023
Boys, pleasant	67.6 (23.1)	0.0035	0.5% (1.0%)	0.4647
Boys, very good	−54.2 (16.8)	0.0013	−3.0% (0.5%)	<0.0001
After educational intervention vs before	−160.7 (70.1)	0.0224	−5.0% (2.0%)	0.0264
**Smell**				
Intercept	685.1 (160.1)	0.0013	43.0% (5.0%)	<0.0001
Girls, unpleasant	115.4(19.8)	<0.0001	1.0% (1.0%)	0.0337
Girls, pleasant	125.6 (22.1)	<0.0001	2.0% (1.0%)	0.0081
Girls, very good	3.0 (15.2)	0.8434	−1.0% (0.5%)	0.003
Boys, unpleasant	63.2 (21.1)	0.0029	2.0% (1.0%)	0.0028
Boys, pleasant	64.7 (22.4)	0.0041	1.0% (1.0%)	0.3316
Boys, very good	−66.1 (17.1)	0.0001	−3.0% (1.0%)	<0.0001
After educational intervention vs before	−160.8 (73.9)	0.0301	−5.0% (2.0%)	0.0187

**β−** Regression coefficient.

**Table 6:** Regression coefficients of the model for evaluating waste weight and percentage from the second course for the numbers of boys and girls expressing pleasant, indifferent or unpleasant judgments on overall aspect, appearance, smell and taste.

	SECOND MAIN COURSE			
	WEIGHT		PERCENTAGE	
Overall aspect	β (SE)	*p*-Value	β (SE)	*p*-Value
Intercept	157.3 (59.6)	0.0229	32.0% (4.9%)	<0.0001
Girls, unpleasant	37.5 (7.5)	<0.0001	1.4% (0.7%)	0.0381
Girls, pleasant	18.2 (8.2)	0.0262	0.4% (0.7%)	0.6167
Girls, very good	−11.9 (5.6)	0.033	−1.4% (0.5%)	0.0047
Boys, unpleasant	35.6 (7.8)	<0.0001	2.5% (0.7%)	0.0006
Boys, pleasant	10.1 (8.9)	0.2617	0.3% (1.0%)	0.7138
Boys, very good	−3.9 (6.1)	0.5102	−2.0% (0.5%)	0.0003
After educational intervention vs before	−41.5 (25.1)	0.0988	−3.0% (2.3%)	0.1667
**Appearance**				
Intercept	169.5 (59.2)	0.0154	33.0% (5.0%)	<0.0001
Girls, unpleasant	37.2 (8.2)	<0.0001	1.6% (0.6%)	0.0348
Girls, pleasant	20.2 (7.2)	0.0052	0.3% (1.0%)	0.6435
Girls, very good	−12.5 (5.6)	0.0257	−1.3% (0.5%)	0.0064
Boys, unpleasant	38.9 (8.5)	<0.0001	3.0% (1.0%)	0.0004
Boys, pleasant	8.3 (8.6)	0.3345	−0.1% (1.0%)	0.8536
Boys, very good	−4.9 (6.1)	0.4128	−2.0% (0.5%)	0.0004
After educational intervention vs before	−36.3 (24.9)	0.1465	−3.0% (2.0%)	0.265
**Taste**				
Intercept	157.3 (59.6)	0.0229	32.0% (5.0%)	<0.0001
Girls, unpleasant	37.5 (7.4)	<0.0001	1.0% (0.6%)	0.0381
Girls, pleasant	18.2 (8.2)	0.0262	0.4% (1.0%)	0.6167
Girls, very good	−11.9 (5.6)	0.033	−1.4% (0.4%)	0.0047
Boys, unpleasant	35.6 (7.8)	<0.0001	2.5% (1.0%)	0.0006
Boys, pleasant	10.1 (8.9)	0.2617	0.3% (1.0%)	0.7138
Boys, very good	−3.9 (6.1)	0.5102	−2.0% (0.5%)	0.0003
After educational intervention vs before	−41.5 (25.1)	0.0988	−3.2% (2%)	0.1667
**Smell**				
Intercept	153.9 (57.9)	0.0223	32.0% (5.0%)	<0.0001
Girls, unpleasant	33.8 (7.4)	<0.0001	1.0% (1.0%)	0.0574
Girls, pleasant	26.3 (7.2)	0.0003	1.0% (1.0%)	0.1309
Girls, very good	−15.3 (5.6)	0.0061	−2.0% (0.5%)	0.0006
Boys, unpleasant	40.2 (7.7)	<0.0001	3.0% (1.0%)	<0.0001
Boys, pleasant	0.9 (8.9)	0.9218	−1.0% (1.0%)	0.2187
Boys, very good	−2.3 (6.1)	0.7044	−2.0% (1.0%)	0.0017
After educational intervention vs before	−36.3 (24.8)	0.1444	−2.6% (2.0%)	0.2524

**β−** Regression coefficient.

**Table 7:** Regression coefficients of the model evaluating waste weight and percentage for the side courses for the numbers of boys and girls expressing pleasant, indifferent or unpleasant judgments on overall aspect, appearance, smell and taste.

	SIDE DISH			
	WEIGHT		PERCENTAGE	
Overall Aspect	β (SE)	*p*-Value	β (SE)	*p*-Value
Intercept	674.8 (122.6)	0.0002	62% (5%)	<0.0001
Girls, unpleasant	12.3 (11.7)	0.2905	−0.7% (0.6%)	0.2869
Girls, pleasant	46.2 (16.8)	0.0064	0.43% (1%)	0.6461
Girls, very good	−16.5 (12.9)	0.2046	−3% (0.4%)	<0.0001
Boys, unpleasant	32.5 (12.8)	0.0115	1.2% (0.6%)	0.089
Boys, pleasant	6.0 (16.3)	0.7135	−0.03% (1%)	0.97
Boys, very good	−54.7 (14.3)	0.0002	−4% (0.5%)	<0.0001
After educational intervention vs before	−24.9 (58.9)	0.6734	−5% (2%)	0.2957
**Appearance**				
Intercept	668.7 (124.3)	0.0002	62% (5%)	<0.0001
Girls, unpleasant	17.2 (12.2)	0.1603	−0.7% (0.6%)	0.2905
Girls, pleasant	32.9 (15.5)	0.0346	0.2% (1%)	0.8074
Girls, very good	−8.9 (12.5)	0.4791	−3% (0.5%)	0.0002
Boys, unpleasant	27.7 (14.4)	0.0546	1.2% (1%)	0.1387
Boys, pleasant	12.1 (15.4)	0.4318	0.07% (1%)	0.9313
Boys, very good	−42.4 (13.8)	0.0024	−3.4% (0.5%)	<0.0001
After educational intervention vs before	−28.7 (62.1)	0.6434	−5% (2%)	0.295
**Taste**				
Intercept	684.7 (122.7)	0.0002	63% (5%)	<0.0001
Girls, unpleasant	20.9 (11.5)	0.0694	−0.3% (0.6%)	0.5838
Girls, pleasant	21.7 (16.9)	0.2013	−1% (1%)	0.2638
Girls, very good	−14.7 (13.1)	0.2627	−3% (0.4%)	0.0002
Boys, unpleasant	27.7 (12.7)	0.0305	1% (1%)	0.1328
Boys, pleasant	18.2 (16.9)	0.2841	0.6% (1%)	0.5144
Boys, very good	−56.4 (14.2)	<0.0001	−4% (0.5%)	<0.0001
After educational intervention vs before	−43.4 (56.8)	0.4459	−6% (2%)	0.176
**Smell**				
Intercept	645.9 (125.8)	0.0003	60% (5%)	<0.0001
Girls, unpleasant	8.8 (11.9)	0.4626	−1.2% (1%)	0.0738
Girls, pleasant	35.4 (15.9)	0.0265	0.1% (1%)	0.8785
Girls, very good	−14.1 (13.3)	0.2882	−3% (0.5%)	0.0003
Boys, unpleasant	42.8 (13.5)	0.0017	2% (1%)	0.0055
Boys, pleasant	6.8 (16.1)	0.673	−0.2% (1%)	0.8028
Boys, very good	−43.4 (13.7)	0.0016	−3.5% (1%)	<0.0001
After educational intervention vs before	−23.9 (59.9)	0.6893	−5% (2%)	0.3014

**β−** Regression coefficient.
